# Parasite–host contact in the Arctic: dispersal behaviour of infective nematode larvae from Svalbard reindeer faeces

**DOI:** 10.1098/rsbl.2024.0715

**Published:** 2025-05-23

**Authors:** Tirza M. Moerman, Kia Karina Tahmin, Stephen J. Coulson, Leif E. Loe, René van der Wal

**Affiliations:** ^1^ Faculty of Environmental Sciences and Natural Resource Management, Norwegian University of Life Sciences, Ås, Norway; ^2^ Department of Arctic Biology, The University Centre in Svalbard, Longyearbyen, Norway; ^3^ Department of Ecology, Swedish University of Agricultural Sciences, Uppsala, Sweden

**Keywords:** parasitology, ecology, laboratory experiment, gastro-intestinal parasitic nematodes, parasite–host interaction, High Arctic

## Abstract

Gastro-intestinal parasitic nematodes are typical pathogens of mammalian herbivores. A key moment of infection by passively ingested nematodes is the contact between infective larvae and the grazing host. Yet, knowledge on dispersal dynamics of larvae infecting wild herbivores in natural environments is limited. We studied the mode and range of lateral larval movement. As study species, we used infective larvae of *Ostertagia gruehneri*—a parasitic nematode that can negatively affect its host, Svalbard reindeer (*Rangifer tarandus platyrhynchus*). In the laboratory, reindeer faecal pats containing larvae were introduced onto soil placed either horizontally or on a slope (10°), mimicking the micro-topography of High Arctic tundra. After four weeks, 939 live nematodes were recorded, of which 23% were in the soil, mostly underneath the faecal pat (20%). The remaining 3% that dispersed away from the pat did so in both sloped and flat soil. We conclude that the larvae were able to actively move from faeces to soil and that subsequent dispersal was limited and not assisted by gravity (slope). These insights reveal potential infection hotspots, providing a glimpse in the complex interplay between parasite and host.

## 1. Introduction

Gastro-intestinal parasitic nematodes are widely prevalent in wild mammalian herbivores [1]. The effect of these parasites on wild hosts can be complex and variable (reviewed in [2]), but a meta-analysis reported that, on average, infection by helminths (including nematodes) reduces the performance of free-living ruminants [3]. Yet, we have limited understanding of parasite dispersal ecology. One of the common relationships concerns passively ingested gastro-intestinal parasitic nematodes with a direct life cycle, which inhabit one definite host and have free-living stages in the environment [4,5]. The larvae develop from eggs into the infective larval stage (L3) in deposited faeces and from there disperse into the surrounding environment to be taken up by their grazing host [4,6]. Herbivores, in turn, can display faecal avoidance behaviour, a strategy believed to minimize parasite infection [7–10]. Here, we focus on the parasite end of this complex interplay between parasite and host. Hence, we study the mode and range of larval dispersal from faecal pats and appraize their influence on the key contact event between parasite and host.

Knowledge on larval movement will help determine parasite distribution patterns and infection hotspots to hosts. The few empirical studies on dispersal of infective larvae from faeces in natural environments have reported minor horizontal distances, with most larvae recorded within a range of 15 cm but exceptionally up to almost 90 cm from the faecal pat [11–14]. Larvae could be passively translocated by raindrops [15], which would spread larvae evenly around the pat. Slopes in the micro-landscape might assist passive larval dispersal, resulting in concentrated larval pools in depression in the micro-landscape. Dispersal could also be driven by active larval movement [6,15,16]. Yet, experimental evidence confirming such active movement away from faeces or aggregation in downslope terrain of parasitic nematode larvae of wild herbivorous mammals is lacking. Improved knowledge of dispersal behaviour and the distribution of infective larvae is crucial for understanding host infection risks.

A natural system of parasitic nematodes and wild herbivorous mammals is present in the High Arctic archipelago of Svalbard (76−81° N and 10−34° E). One of the dominant parasitic nematodes of Svalbard reindeer (*Rangifer tarandus platyrhynchus*) [17] is *Ostertagia gruehneri* (Ostertagia: Trichostrongylidae) [1]—a species with a direct life cycle and of which infective larvae are passively ingested [18,19]. An anthelmintic experiment revealed that *O. gruehneri* can depress body condition and fecundity in Svalbard reindeer [20], with modelling suggesting that this could regulate reindeer population size [21]. The reported impact of this parasite on a wild mammalian host makes this a particularly interesting and relevant system to study the key contact event between parasite and host. Yet, dispersal abilities of larvae in Arctic environments have not been studied.

In this study, we test (i) if larvae of *O. gruehneri* can display active movement from the faecal pat, (ii) if a micro-topographical slope assists larval dispersal, which could lead to aggregation and (iii) what the potential dispersal distances are on flat and sloped terrain.

## 2. Material and methods

### (a) Experimental setup

**Figure 1.**
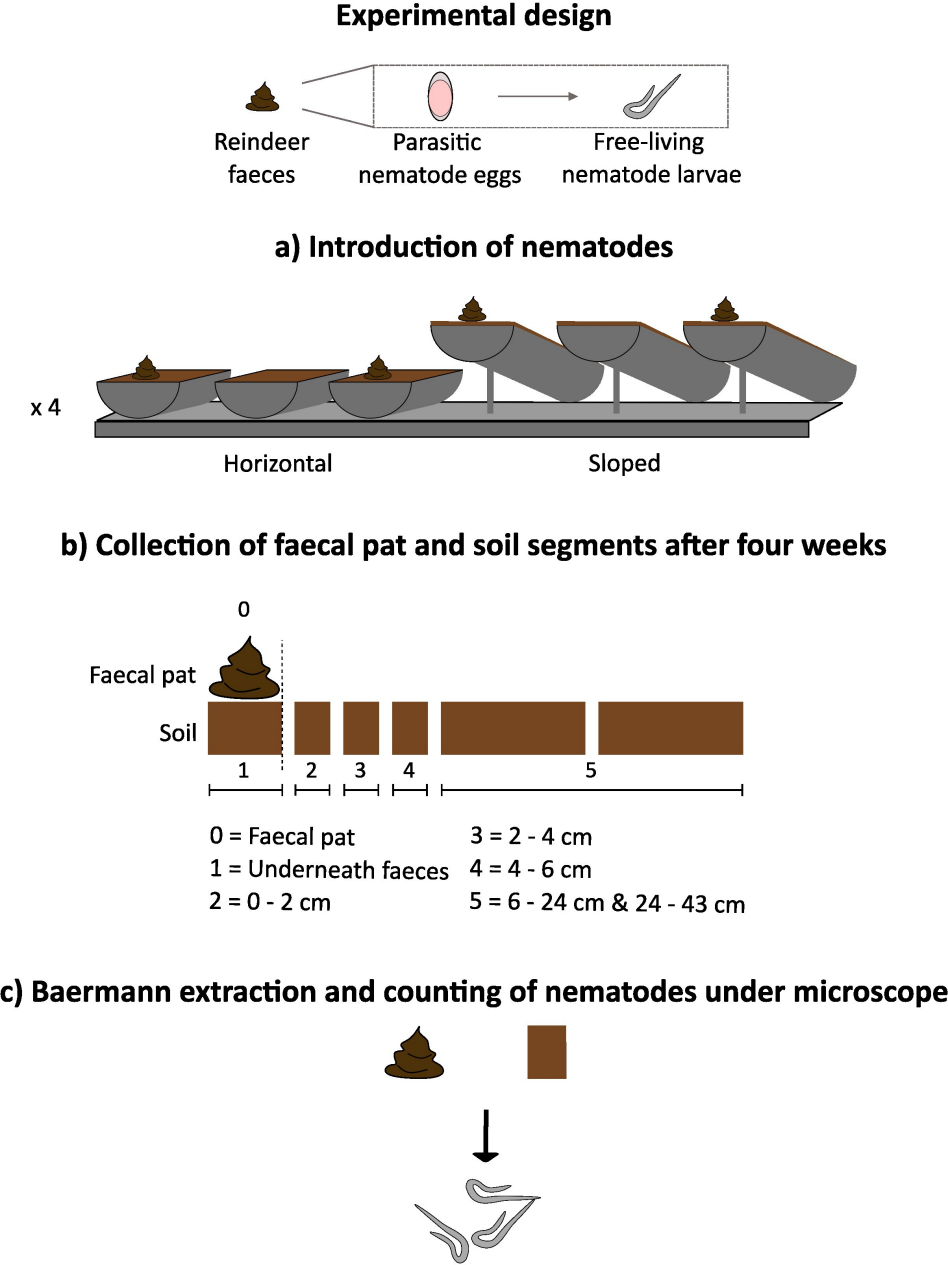
Experimental set-up in a climate chamber to study the mode and range of lateral movement of parasitic nematode larvae of *O. gruehneri*. (a) Fresh homogenized reindeer faeces, containing parasitic nematode eggs, were placed on soil in horizontal or sloped (10°) half-pipes. A third set of half-pipes, added to both treatments to conduct soil moisture measurements without compromising possible nematode movement, contained soil without a faecal pat. In total, we had four plates with this identical set-up. (b) After four weeks (allowing nematode larvae to develop from eggs and possibly disperse), faeces and soil (cut into horizontal segments) were collected. (c)The nematode larvae were extracted from faeces and soil using Baermann funnels and counted under a microscope.

A laboratory set-up was designed to test the lateral dispersal capabilities of infective parasitic nematode larvae (figure 1). The four-week experiment was conducted indoors at research facilities of the University Centre in Svalbard in Longyearbyen, Svalbard.

#### (i) Collecting soil and faeces

Top soil was collected from Adventdalen (Svalbard) and sterilized by autoclaving (12 l high-pressure laboratory autoclave, 125°C for 25 min). We perceived the soil to be clean after autoclavation, although we found one live nematode (most likely a free-living soil nematode due to contamination) across six sub-samples of the autoclaved soils (each weighing ± 20 g). We used soil as substrate because we could autoclave this while maintaining the natural structure, but we acknowledge that this reduces the micro-habitat complexity that is present in natural environments.

Fresh reindeer faeces were collected in Adventdalen and Bjørndalen, Svalbard (valleys nearby the research facilities) in mid-August 2023. The faeces were collected from the ground, while care was taken to avoid inclusion of soil or plant material. The faeces were stored in buckets covered with punctured plastic foil at 5–6°C until the start of the experiment. Using a double-centrifuge method with a saturated salt solution (electronic supplementary material, S1a), respectively, 34, 71 and 132 eggs of *O. gruehneri* were counted in three random 1 g sub-samples of faeces. The counts of these three random sub-samples confirmed the presence and dominance of *O. gruehneri* eggs in the faeces, with large variation in abundance between samples (corresponding to previous observations; [17]).

#### (ii) Experiment

The experiment was conducted in a climate-controlled room (14–15°C; 24 h of artificial light). At the start of the four-week experiment (4 September 2023), faeces containing *O. gruehneri* eggs were carefully but extensively mixed to homogenize egg density (as in [11]) and round faecal pats (50 ± 1 g) were formed. The faeces were placed on mixed autoclaved soil (210 ± 1 g) in plastic half-pipes (made of polypropylene pipe cut in half) of approximately 50 cm long, 5 cm diameter and closed with an end plate. To test for active dispersal behaviour and gravity-assisted dispersal on slight slopes, the half-pipes were placed in two different slope treatments that mimicked local micro-topography in the landscape: horizontal (0°) and sloped (10°). An angle of 10° is a commonly occurring micro-topographical slope in Svalbard [22] and was chosen because it provides some gravitational force to possibly assist larval dispersal, while avoiding soil from moving down the slope. A third set of half-pipes consisted of soil without a faecal pat, but also placed horizontally or at a slope; these were used to monitor moisture levels during and at the end of the experiment. Plastic plates were used to support the construction of these horizontal and sloped half-pipes, with on one plate, two horizontal and two sloped half-pipes with faecal pats, and one horizontal and one sloped half-pipe without faecal pat for moisture measurements. In total, we had four plates with this identical setup (figure 1a).

During the experiment, all half-pipes were moistened daily with a plant hand-sprayer that produced a low-impact mist. The addition of moisture was designed to create damp soil and faeces, but without causing water run-off. This method resulted in fluctuating moisture conditions, with damp conditions right after spraying but drier conditions before the next watering event, and an overall decreasing trend (electronic supplementary material, S1b, figure S1). Except for the daily watering events, the treatment half-pipes with faecal pats were left undisturbed for four weeks. This duration allowed infective larvae, developing from eggs in the faeces, to complete development and potentially disperse.

#### (iii) Extracting and counting nematodes

After four weeks, four pipes (two horizontal and two sloped) were collected each day over a period of 5 days (starting at 30 September 2023). We subsequently collected the faecal pat, the soil underneath the faecal pat and soil segments along the half-pipe (0−2, 2−4, 4−6, 6−24 and 24−43 cm relative to the edge of the faecal pat, figure 1b) to detect both active and possible slope-assisted dispersal. All samples were weighed before extracting. Due to a sampling error, data are missing for five samples, one sample underneath the pat and four samples in segment 6−24 cm, but this is believed not to impact the overall results (electronic supplementary material, table S1).

Nematode extraction from the soil and faecal samples was done immediately after collection. We used a modified Baermann method (with a paper filter on a metal wire mesh support in a plastic container; electronic supplementary material, S1c), a method based on the active movement of nematodes out of a sample [23]. The study was designed with high spatial resolution close to the pat to detect active dispersal and lower spatial resolution further away from the pat to capture possible assisted dispersal. All nematodes with observed movement within 10 s of inspection were counted and presumed to be *O. gruehneri*. This assumption was made because of the careful collecting of fresh faeces, the autoclaving of soil and because of the high prevalence and dominant egg output of *O. gruehneri* in reindeer summer faeces, greatly outnumbering *Marshallagia marshalli* (the other major gastro-intestinal parasitic nematode of Svalbard reindeer [17]). In reindeer faeces collected in August, we expect a ratio of approx. 150 *O*. *gruehneri* to 1 *M*. *marshalli* [17]. In the case of similar dispersal behaviour of the two nematodes (both species are passively ingested parasitic nematodes [18]), we would expect that less than 1% of all nematodes along the entire tray might belong to *M. marshalli*, and we therefore deem the potential influence of this other species negligible. Because of this strong *a priori* expectation and logistical constraints, no further taxonomic identification was conducted. Counting of nematodes was randomly divided between two counters. A subset of samples was counted by both observers, but only one of these ‘double’ counts, randomly selected, was used in the analysis. Observer bias in nematode count was checked for but not found (see under statistical analysis).

### (b) Statistical analysis

All analyses were done in R v. 4.4.1 [24]. Data exploration [25] indicated no clear outliers and many observations with zero nematode recordings. Due to the experimental design, the soil segments collected at various distances relative to the pat differed in length and thus weight (figure 1b). Segment weight was not included in the model, but we checked for possible patterns in the plots of model residuals against weight (see below). In addition, for the segments 6−24 cm, nematodes were found in only one soil sample. The segments 6−24 and 24−43 cm were therefore combined in the statistical analysis.

To test for slope-assisted dispersal and potential dispersal distances, we modelled nematode count in each segment as a function of the covariates slope (categorical with two levels), segment (categorical with six levels) and their interaction. To account for structure in the data (multiple observations per half-pipe and multiple half-pipes per plate; figure 1a), we applied a generalized linear mixed effect model (GLMM), using the glmmTMB package [26], declaring half-pipe nested within plate as a random effect. Nematode counts were modelled assuming a negative binomial distribution and a logarithmic link function [27]: nematode recordings ~ slope + segment+slope * segment + (1│plate / half-pipe). Model assumptions were verified using the package DHARMa [28] and by plotting Pearson’s residuals against fitted values and all covariates in-and-excluded from the model (e.g. observer and segment weight). These plots did not indicate deviating patterns (electronic supplementary material, figure S2). To additionally validate the accuracy of the model, datasets were simulated using the fitted model with the simulate function (R base [24]). The number of zeros and the maximum nematode recordings from these simulated datasets were compared with the observed values and verified that the model fitted our data properly. The summary outputs from the GLMM were presented using the tab_model function of the package ‘sjPlot’ [29]. Estimated means based on the fitted model were calculated using the predict function (R base [24]) and visualized using the package ‘ggplot2’ [30].

## 3. Results and discussion

**Figure 2.**
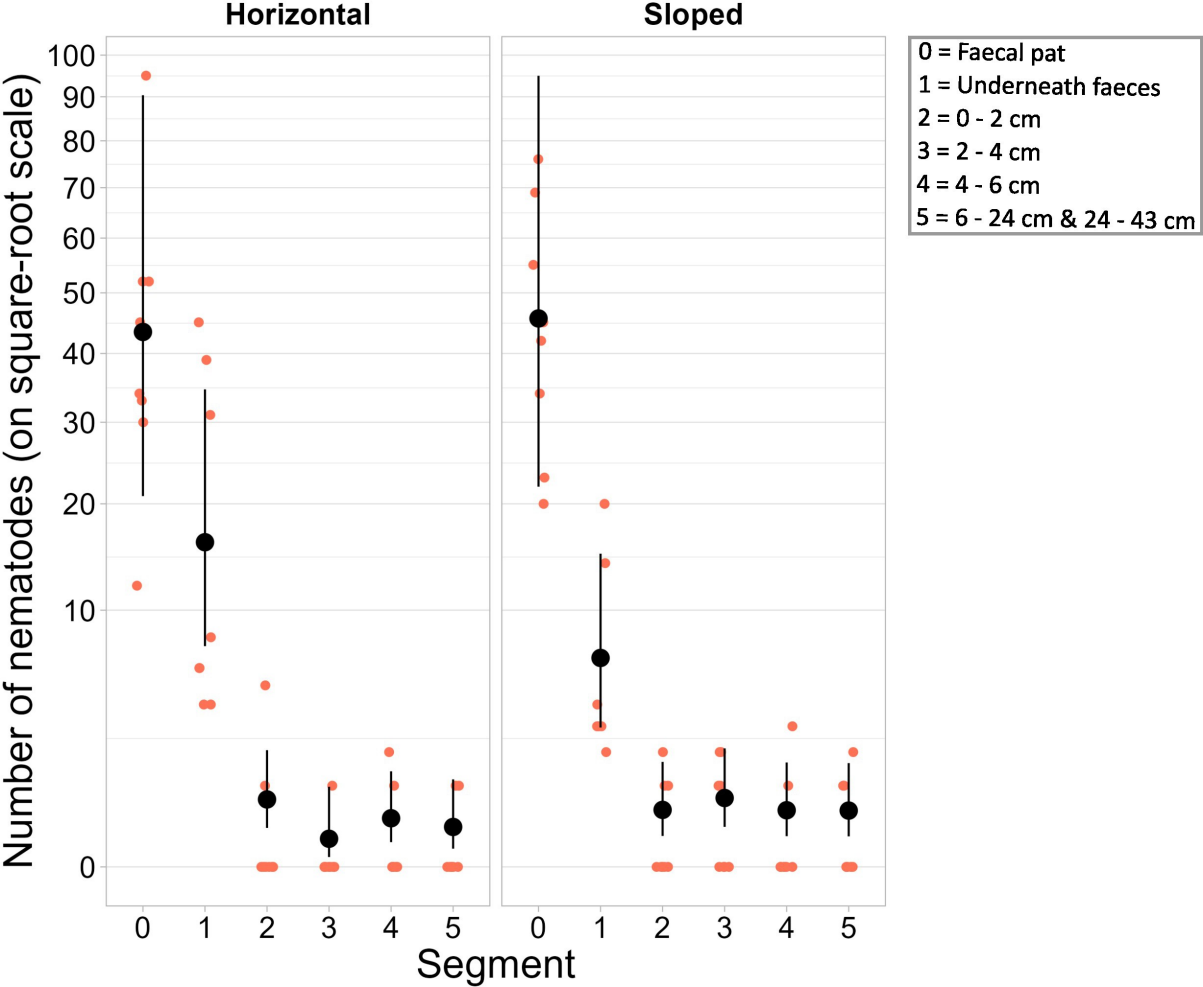
The number of *O. gruehneri* larvae for each segment (0 = in faecal pat, 1 = soil under faecal pat and 2−5 = soil at increasing distances from pat, see also figure 1) and slope treatment (horizontal = slope of 0° and sloped = slope of 10°), with *y*-axis on a square-root scale. Raw data are displayed as small red-brown circles. The predicted means (larger black circles) with their 95% CIs are back-transformed estimates from the GLMM for the average plate and half-pipe.

After the four-week duration of our experiment that mimicked micro-topography in the tundra, a total of 939 live nematode larvae were recorded. Of these, 23% were in the soil, demonstrating that the larvae can move unaided from faeces into soil. Only a few of the larvae found in soil had moved away from the pat (3% of total nematode recording; figure 2, electronic supplementary material, table S1). Recording distance was not affected by the slope treatment (*p* = 0.9, table 1), indicating that larval dispersal was not assisted by the slight (10°) slope.

**Table 1.** Summary outputs from the GLMM, predicting the number of *O. gruehneri* larvae as a function of interacting effects of slope (horizontal and sloped) and segment (in faeces, under faeces and four soil samples at increasing distance from the faecal pat). Half-pipe within plate was included as a nested random effect. The reference level is segment 0 (faecal pat) and horizontal.

predictors	explanation	log-mean	SE	*95%* ***CI***	*p-*value
(intercept)	faecal pat and horizontal	3.77	0.25	3.29 to 4.25	<0.001
slope [sloped]		0.05	0.34	−0.62 to 0.72	0.884
segment 1	soil under faeces	−1.00	0.33	−1.65 to −0.35	0.003
segment 2	soil 0–2 cm	−4.14	0.52	−5.16 to −3.12	<0.001
segment 3	soil 2–4 cm	−5.89	1.05	−7.95 to −3.84	<0.001
segment 4	soil 4–6 cm	−4.80	0.66	−6.08 to −3.51	<0.001
segment 5	soil 6–43 cm	−5.19	0.77	−6.71 to −3.67	<0.001
slope [sloped] × segment 1		−0.93	0.49	−1.88 to 0.02	0.056
slope [sloped] × segment 2		−0.39	0.78	−1.93 to 1.15	0.622
slope [sloped] × segment 3		1.74	1.17	−0.54 to 4.03	0.135
slope [sloped] × segment 4		0.25	0.88	−1.47 to 1.98	0.773
slope [sloped] × segment 5		0.64	0.97	−1.27 to 2.54	0.512

random effects					
σ^2^ (residual variance)					0.38
τ_00 Halfpipe:Plate_ (random intercept variance)					0.08
τ_00 Plate_ (random intercept variance)					<0.01
ICC (intra-class correlation coefficient)					0.18
marginal R^2^ (fixed effect)/conditional R^2^ (fixed+random effect)					0.897/0.916
dispersion parameter					2.8

### (a) Larval hotspots underneath faecal pats

Of the nematodes in the soil, the majority were recorded in the soil underneath pats (20% of total nematodes recorded; figure 2, electronic supplementary material, table S1), indicating this area as a possible infection hotspot. This adds support to the suggestion for soil to be a possible reservoir for infective larvae [31–33]. The area underneath pats may be protected against adverse conditions, such as peaks in low and high temperatures or high evaporation rates. Water is believed to play an important role for the ability of larvae to escape from faeces [32,34]. Here, the fine mist created by our plant hand-sprayer appeared to be sufficient for these larvae to move unaided out of the faeces. Measurements on the moisture content at the end of the experiment (using half-pipes with only soil) did not indicate moisture differences in moisture patterns between sloped and horizontal half-pipes (electronic supplementary material, S1b, figure S1).

### (b) No evidence for larval aggregation in the lower terrain

We recorded 30 nematodes (3% of total nematode recording) in the soil segments along half-pipes further away from the faecal pat, including the segment 24−43 cm (figure 2, electronic supplementary material, table S1). These observations were independent of slope (table 1). As a consequence of our experimental design, soil segments varied in volume, possibly introducing an extraction bias, implying that comparison between samples of different sizes and substrates should be done with care. However, all soil samples were perceived small enough to warrant good extraction, and any possible bias will be similar for sloped and horizontal trays. The results here suggest that the nematodes that dispersed laterally were capable of doing so actively and that a slope did not assist larval dispersal. Although we cannot rule out that natural water flow might play a role in assisted dispersal in sloped terrain, the observations here do not provide evidence for the presence of downslope larval aggregation. Instead, it suggests that larvae remain under or very near to the faecal pat.

### (c) The majority of larvae still in the faeces

Despite many nematodes in the soil, the majority of nematodes were found in the faeces (76% of total nematode recording, table 1, figure 2). High numbers of larvae in the faecal pat may not be surprising as larvae have been found in 1-year-old *Rangifer* spp*.* faeces on the tundra [10,35], which corresponds with previous suggestions of faeces as another larval reservoir [32,36,37]. Here, the combination of high larval counts in the faeces and the demonstrated ability of larvae to move from faeces into soil under minimum moisture conditions suggests the potential of gradual larval release from faeces to the Arctic tundra. Dispersal dynamics, such as gradual versus mass larval release (also discussed in [32]), will have implications for transmission patterns. While mass larval release would result in short spells of high infection risks, gradual larval release can result in lower larval abundance at once, but spread infection risk over much longer time periods.

### (d) Should I stay or should I go?

Insights on larval dispersal allow us to hypothesize about (optimal) strategies for larvae to increase their chances to be ingested by reindeer. Substantial larval presence underneath the pat suggests that larvae may benefit from dispersing to soil underneath the pat—a location clearly used by the host—and then opt for a waiting strategy [16] to be ingested. The pat might be flushed away by run-off (during e.g. snowmelt) or be consumed by dung-flies (e.g. *Scatophaga* spp.). Faecal avoidance behaviour might also decrease as faeces age, as is shown in domesticated sheep [38]. All this could remove possible warning signals that the faecal pat may display and result in contact between parasite and host at locations where faeces were initially deposited. However, Svalbard reindeer can show avoidance towards plots contaminated with faeces from the previous year [10], which coincides with observations of *O. gruehneri* larvae in 1-year-old faeces [10,35]. Consequently, the contact between parasite and host, and infection risk may span over long time periods, potentially even multiple years. Our observation of nematodes in soil along the tray point in the direction that some larvae opt for a more active dispersal strategy [16]. These larvae may move out of the pat area avoided by reindeer and thereby increase their chance to be ingested. At the same time, these larvae risk depleting energy reserves, as infective larvae are believed not to feed [39–41]. These larvae may also end up in a micro-landscape not used by reindeer, since plant biomass and species composition vary over small spatial scales [42] and grazing tracks such variation in the landscape [43]. Altogether, the key contact event between parasite and host is complex, and insights on larval dispersal provide a glimpse into this interplay.

### (e) Concluding remarks

In conclusion, we have demonstrated that under conditions mimicking Arctic soil and micro-topographical slope, larvae of *O. gruehneri* were able to actively move out of faecal pats. Within the four-week study period, dispersal occurred mainly to soil beneath the pat. Very few larvae were recorded in soil further away from the faecal pat, and sloped terrain did not assist them to do so. This awareness suggests (longer-term) infection hotspots very near to faecal pats rather than aggregations of infective larvae in depressions in the tundra. We did not test how moisture affects larval dispersal, although larval dispersal can be influenced by environmental factors such as precipitation [34,44]. Understanding how moisture and other environmental conditions influence larval dispersal would be highly relevant in the light of the rapidly changing climatic conditions across the Arctic [45–47], and experimental set-ups as described here could be used as a starting point. Altogether, fundamental knowledge on larval dispersal enhances our understanding of larval distribution in the landscape and the key contact moment between parasite and host.

## Data Availability

All data and R scrips can be found as electronic supplementary material, together with a meta-data file. Supplementary material is available online [48].
